# Performance of preschool and schoolchildren on the ProNOH protocol: macrostructure aspects

**DOI:** 10.1590/2317-1782/20232022245en

**Published:** 2023-11-20

**Authors:** Natalia Freitas Rossi, Ana Carolina Xavier, Kriscia Gobi Rosa, Célia Maria Giacheti

**Affiliations:** 1 Programa de Pós-graduação em Fonoaudiologia, Faculdade de Filosofia e Ciências de Marília, Universidade Estadual Paulista “Júlio de Mesquita Filho” – UNESP - Marília (SP), Brasil.; 2 Instituto Nacional de Ciência e Tecnologia sobre Comportamento, Cognição e Ensino – INCT-ECCE, Universidade Federal de São Carlos – UFSCar - São Carlos (SP), Brasil.; 3 Departamento de Fonoaudiologia, Faculdade de Filosofia e Ciências de Marília, Universidade Estadual Paulista “Júlio de Mesquita Filho” – UNESP - Marília (SP), Brasil.

**Keywords:** Narration, Language, Child Development, Language Tests, Students

## Abstract

**Purpose:**

To investigate if the narrative score of the ProNOH protocol allows for discriminating age groups, as well as its relation with the global coherence level of the story. The performance of preschool and schoolchildren on the macrostructure aspects.

**Methods:**

Participants were 97 preschoolers and schoolchildren with typical language development, aged between five and 12 years old, and both sexes who attended public schools. The “Protocolo de Avaliação da Narrativa Oral de História (ProNOH)” (Protocol for the Evaluation of Oral Storytelling) was applied and the narrative score in the macrostructure dimension was calculated with story grammar elements. These same story elements were used to obtain the global coherence level of the story, as proposed by Spinillo and Martins (1997).

**Results:**

A statistically significant difference was found between age groups, mainly between the borderline ages of 5-6 years, 7-8 years, 9-10 years, and 11-12 years. Positive and statistically significant correlations were found between the narrative score and global coherence and age, as well as between the narrative score and global coherence.

**Conclusion:**

The protocol proved to be useful for identifying the repertoire of typical story grammar elements as an objective measure that differs in oral narrative across age groups. The results also indicate that the narrative score can provide an idea about the global coherence of the story, although this value does not replace a specific analysis.

## INTRODUCTION

The oral narrative is one of the discursive skills and represents one of the most complex levels of language organisation with its cognitive scheme of mental representation, with rules and structural elements that are peculiar and confer specificities that allow distinguishing a conversation from a narrative, as well as distinguishing the different types of narrative; autobiographical or story^(1)^.

The story-type narrative is constituted by structural elements typical of story grammar, which are considered part of the macrostructure aspects of the narrative. The macrostructure dimension can be considered the cognitive, executive component that is responsible for the general organisation of the plot, for sustaining the theme, and for maintaining information throughout the narrative to constitute a logical and coherent plot. In turn, the microstructural dimension can be considered the dimension of a linguistic nature, which comprises the phonology of words, the organisation and syntactic complexity of utterances, vocabulary, as well as the use of cohesive resources. Both macro and microstructural aspects have been adopted in studies as indicators of the development of storytelling skills^(2-4)^.

Children with deviant language development may have both comprehension and storytelling difficulties^(5,6)^. The effects of cognitive-linguistic impairments on narrative skills in these children are described as heterogeneous and variable depending on the etiology and other related factors^(7).^

In Language Development Disorder (LDD), the changes seem to be more centred on the microstructural aspects of narrative^(5)^. On the other hand, impairments in the macrostructure of the narrative have been quite explicit in cases where language difficulties are associated with genetic syndromes that occur with Intellectual Development Disorder (IDD), as well as in Autism Spectrum Disorder (ASD), as pointed out in a review study^(7)^ and Attention Deficit Hyperactivity Disorder (ADHD)^(8,9)^.

Oral storytelling has been considered an important link between oral and written language skills due to the sharing of cognitive processes that subsidise language modalities, such as executive functions^(10)^.

In addition to executive functions, the oral storytelling task also reflects the integration of structural components of language (e.g., morphosyntax, phonology) and content (semantics), which are applied in functional communication (pragmatics), managed by the typical structural elements of story grammar^(10)^.

Regarding the methods of investigating performance in oral narrative, it is possible to find different proposals for collection and analysis in the literature. The use of a sequence of images to elicit stories, mainly through books, is considered a favourable resource, since it helps in the elaboration of the narrative scheme of the story and allows the evaluator, to some extent, a certain control of the content being narrated, which is not possible in personal narratives^(11,12)^.

Among the most cited picture books in the literature as a resource for eliciting narrative, the book “Frog, where are you?”^(13)^ is widely referenced. Its structure is advantageous for cross-cultural studies, since it has only illustrations, without writing, with a level of complexity sufficient for an analysis of important aspects in narrative studies, such as temporal, causal, and spatial relationships between events^(14)^. The book “Frog, where are you?”^(13)^ has an internal organisation that favours the organisation of complex levels of the narrative story scheme and allows for the analysis of different age groups, including adults^(14,15)^.

This book^(13)^ has been used both in studies conducted with individuals with typical and atypical language development^(4,16)^ and also used as part of protocols to propose a system for analysing narrative performance, based on macro- and microstructural elements^(17-20)^.

The scarcity of specific and standardised sequential images to investigate oral storytelling is a reality in the Brazilian clinical and research scenario. In this context, the “Protocol for the Evaluation of Oral Storytelling (ProNOH)”^(19)^ was developed as a systematic proposal for analysing the macro and microstructural aspects of the narrative elicited from the book “Frog, where are you?”^(13)^. The protocol presents instructions for analysing narrative performance through a score assigned by the evaluator based on the typical story elements, which allows for determining the narrative score, which is generated from the macro-structural dimension and criteria related to the microstructural aspects, thus constituting a tool that can be used both for evaluation purposes and for therapeutic monitoring (evolution) of narrative skills^(19)^.

Therefore, the present study aimed to investigate whether the narrative score obtained through the application of the “Protocol for the Evaluation of Oral Narrative History (ProNOH)”^(19)^ allows discrimination against the age groups studied, as well as its relationship with the level of global coherence of the story.

## METHODS

This study is part of a broader project approved by the Research Ethics Committee of the Universidade Estadual Paulista “Júlio de Mesquita Filho” (UNESP) - Câmpus de Marília (process no. 1105/2014; 5.391.347). Parents and/or guardians authorised the children's participation by signing the Informed Consent Form (ICF),

The sample consisted of 97 participants (47 males and 50 females) aged between five and 12 years (M=8.46, SD=2.34), attending kindergarten to primary schools (1st to 6th grade) of three public schools in a municipality in the interior of the State of São Paulo.

The inclusion criteria adopted were as follows: (a) no previous history and/or persistent language/learning disorders, as well as syndromic medical conditions, psychiatric and/or neurological disorders; (b) no sensory disorders (auditory and/or visual); (c) attending public and regular education; (d) having been indicated by the teacher as a student with adequate school performance. Table 1 summarises the frequency distribution by age group and sex of the participants.

**Table 1:** Distribution of the sample in age groups

Age group	Frequency	Gender (%)	
		Male	Female
5	13	4 (30.7)	9 (69.2)
6	12	9 (75.0)	3 (25.0)
7	13	8 (61.5)	5 (38.5)
8	12	7 (58.3)	5 (41.7)
9	11	3 (27.3)	8 (72.7)
10	11	3 (27.3)	8 (72.7)
11	12	5 (41.7)	7 (58.3)
12	13	8 (61.5)	5 (38.5)

The oral narrative was collected and analysed for macrostructure aspects using the book “Frog, where are you?”^(13)^ and according to the instructions and criteria for analysis and scoring proposed in the “Protocol for the Evaluation of Oral Narrative History - ProNOH”^(19)^.

The average time spent using the protocol was: (a) 3.5 to 5.0 minutes for the narration; (b) 50 minutes for transcribing the narrative and; (c) 30 minutes for assigning the scores and summing the partial and global scores of the narrative.

To obtain the narrative score, scores were assigned according to the presence of information distributed in five structural story categories as follows: setting, theme, plot, challenges, and resolution, as proposed by Rossi, Rosa and Giacheti^(19)^ as well as the partial and global score was determined.

To classify the level of global coherence of the story, the proposal by Spinillo and Martins (1997)^(21)^ was adopted to analyse the global coherence of the story, and it considers the structural elements as indicators of coherence, the same previously identified to establish the narrative score by the ProNOH protocol. The items analysed to establish the level of coherence were: the maintenance of the characters throughout the narrative; the theme maintenance around the main event and secondary events that articulate with the main event; and the relationship between the narrated events connected and the characters, with the presence of an outcome that ends and concludes the story concerning the main event. Based on these factors, the stories were categorised into four increasing levels of complexity (Level 1, Level 2, Level 3, and Level 4), which inform the degree of overall coherence of the story.

### Statistical analysis

Descriptive statistics were analysed to obtain the mean, standard deviation, and 25th, 50th, and 75th percentiles of the partial and global narrative score. In the case where the Shapiro-Wilk normality test indicated that the data adhered to normality, the test of equality of means was performed to verify possible differences between the means in the Analysis of Variance (ANOVA) with one factor with multiple comparisons. Post-hoc analysis of multiple comparisons was conducted with Tukey's test when the ANOVA result indicated statistical significance (*p*<0.05).

Pearson's correlation statistical test was used to investigate the possible correlation of the narrative score (total score) with age and the level of global coherence of the story.

The significance level adopted in the study was 0.05%. Data analysis was performed using the Minitab 1.6 programme.

## RESULTS

Table 2 shows the means, standard deviations, and percentiles of the narrative score of the age groups from five to 12 years established from the ProNOH in the macrostructure aspects.

**Table 2:** Means, standard deviations, and percentiles of the age groups in ProNOH-macrostructure aspects

		Age							
		5 years	6 years	7 years	8 years	9 years	10 years	11 years	12 years
Scenario	Mean	2.5	3.4	4.0	4.6	4.6	5.2	5.7	6.0
	SD	0.5	0.5	0.7	0.9	1.0	0.7	0.9	1.0
	Percentile 25	2.0	3.0	3.5	4.0	4.0	5.0	5.0	5.0
	Percentile 50	2.0	3.0	4.0	4.5	4.0	5.0	5.0	6.0
	Percentile 75	3.0	4.0	4.5	5.0	5.0	6.0	6.7	7.0
Theme	Mean	0.9	2.0	2.0	2.0	2.0	2.0	2.0	2.0
	SD	0.6	0.0	0.0	0.0	0.0	0.0	0.0	0.0
	Percentile 25	0.5	2.0	2.0	2.0	2.0	2.0	2.0	2.0
	Percentile 50	1.0	2.0	2.0	2.0	2.0	2.0	2.0	2.0
	Percentile 75	1.0	2.0	2.0	2.0	2.0	2.0	2.0	2.0
Plot	Mean	1.6	3.7	4.7	4.7	5.2	5.7	5.5	6.3
	SD	0.6	0.9	0.7	0.8	1.2	0.6	0.5	1.1
	Percentile 25	1.0	3.0	4.0	4.0	4.0	5.0	5.0	5.5
	Percentile 50	2.0	4.0	5.0	4.5	6.0	6.0	5.5	6.0
	Percentile 75	2.0	4.0	5.0	5.0	6.0	6.0	6.0	7.0
Problem-solving	Mean	0.8	1.1	1.1	1.3	1.6	2.0	2.0	2.0
	SD	0.4	0.3	0.4	0.5	0.5	0.0	0.0	0.0
	Percentile 25	1.0	1.0	1.0	1.0	1.0	2.0	2.0	2.0
	Percentile 50	1.0	1.0	1.0	1.0	2.0	2.0	2.0	2.0
	Percentile 75	1.0	1.0	1.0	2.0	2.0	2.0	2.0	2.0
Challenges	Mean	2.2	4.8	4.2	5.4	5.4	6.0	6.0	7.5
	SD	0.4	1.0	0.7	0.9	1.1	0.8	0.9	0.9
	Percentile 25	2.0	4.0	4.0	5.0	4.0	5.0	5.0	6.0
	Percentile 50	2.0	4.5	4.0	5.5	6.0	6.0	6.0	8.0
	Percentile 75	2.5	5.7	5.0	6.0	6.0	7.0	7.0	8.0
Linguistic markers	Mean	0.38	0.41	0.69	1.16	0.45	0.81	0.75	0.4
	SD	0.50	0.51	0.63	0.83	0.68	0.60	0.45	0.6
	Percentile 25	0.0	0.0	0.0	0.25	0.0	0.0	0.25	0.0
	Percentile 50	0.0	0.0	1.0	1.0	0.0	1.0	1.0	0.0
	Percentile 75	1.0	1.0	1.0	2.0	1.0	1.0	1.0	1.0
Total (Narrative score)	Mean	8.5	15.4	16.8	19.2	19.4	21.8	21.9	24.1
	SD	1.8	1.8	1.3	1.4	2.4	1.6	1.5	2.2
	Percentile 25	7.0	14.0	16.0	18.0	18.0	21.0	21.0	22.0
	Percentile 50	8.0	15.0	16.0	19.0	18.0	22.0	21.5	23.0
	Percentile 75	10.0	16.7	18.0	20.0	21.0	23.0	23.7	26.0

Caption: SD = Standard Deviation

**Figure 1:**
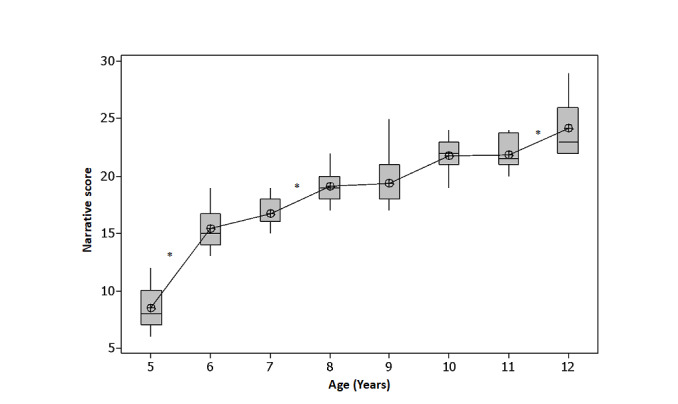
Narrative score (total score) in ProNOH-macrostructure aspects in the age groups

A statistically significant difference was found in the comparison of the narrative score means in the age groups using ANOVA (F=95.21; *p*<0.000). The post-hoc analysis using Tukey's test showed a statistically significant difference between the borderline ages of 5-6 years, 7-8 years, 9-10 years, and 11-12 years, with the following mean representations: 5 years < 6 years = 7 years < 8 years = 9 years < 10 years = 11 years > 12 years (Figure 1).

Caption: (*) *p*<0.05 in the comparison between the age group in the post-hoc analysis by Tukey's test

The frequency distribution of the classification of the overall coherence level of the story (Table 3) indicated that the lowest coherence level (Level 1) was only found in the five year old age group. The majority of the six-seven years old participants showed Level 3 of coherence. The eight years old group was equally divided between level 3 and 4 and above nine years old, the majority presented level 4 of overall coherence.

**Table 3:** Frequency of coherence levels found in the age groups

**Age group**	**Coherence level**			
	**Level 1**	**Level 2**	**Level 3**	**Level 4**
5 years (N=14)	11	3	0	0
6 years (N=12)	0	2	10	0
7 years (N=13)	0	0	13	0
8 years (N=12)	0	0	8	4
9 years (N=11)	0	0	4	7
10 years (N=11)	0	0	2	9
11 years (N=12)	0	0	1	11
12 years (N=13)	0	0	0	13

**Figure 2:**
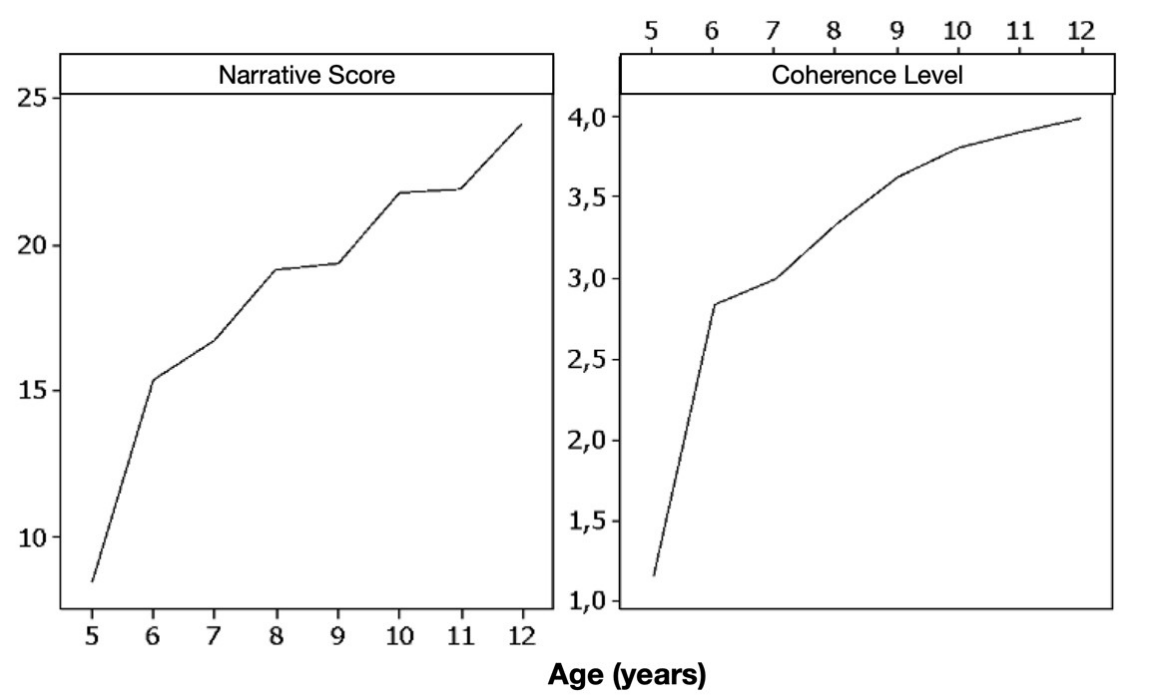
Distribution of the narrative score and level of story coherence according to the age of the participants

Pearson's correlation test found a statistically significant and positive correlation between age and narrative score (r=0.880 and *p*=0.000) and between age and overall narrative coherence level (r=0.786, *p*=<0.001). Figure 2 shows the distribution of narrative score and level of coherence as a function of age.

Caption: (*) *p*<0.05. Pearson's correlation test

**Figure 3:**
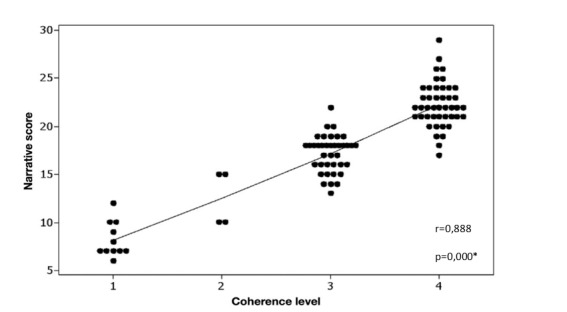
Distribution of the narrative score according to the level of global coherence of the participants' story

Pearson's correlation test also found a statistically significant and positive correlation between the narrative score and the level of global coherence of the story (r=0.888; *p*=0.000). Figure 3 shows the distribution of the narrative score as a function of the participant's level of global story coherence.

## DISCUSSION

This study aimed to investigate whether the narrative score obtained through the application of the “Protocol for the Evaluation of Oral Storytelling (ProNOH)”^(19)^ allowed the age groups studied to be discriminated, as well as its relationship with the level of global coherence of the story. This protocol was proposed to systematise the oral storytelling elicitation resource and the analysis of story elements, thus proposing a scoring system that would allow the performance in oral storytelling to be objectively measured from the macro and microstructural aspects. In this study, the data obtained are specifically focussed on the macrostructure aspects.

Regarding the performance of preschoolers and schoolchildren in the 5-12 age groups, it was found that in the “scenario” element, the attribution of proper names to the characters (boy, dog, and frog) was more common in the stories narrated by schoolchildren from 8-9 years old. Also, younger children under eight years old, especially preschoolers, had more difficulty perceiving the existence of a temporal passage in the story that is represented by various graphic elements such as the “moon”, which can be seen through the bedroom window (scenes 1 and 2), the boy and the dog sleeping in bed (scene 2) and the absence of the “moon” element with the light entering the room (scene 3). It is noticeable in the illustration of scene 3 that the lightness is represented by the decrease of the shading on the wall where the window is arranged.

Regarding the item “theme”, it was found that although the children in the 6-7 age group were able to identify the existence of a problem or complication in the story, making the initial event explicit, they had difficulty mentioning the existence of an internal plan of the main character (the boy's decision to look for the frog) to solve the problem. The proposition of a plan of action, as well as the use of linguistic elements that make this plan explicit (“the boy decided to look for the frog”), was more present in the narratives of groups 8-9 years and especially from 10 years onwards.

The succession of temporally organised actions (represented in scenes 4 to 18 of the book) that show the character's various attempts to solve the problem constitute the plot of the narrative. In analysing this item, it was possible to observe that the number of actions narrated by the children was different in the age groups. Qualitatively, it was also possible to identify differences between the groups. The main differences were seen in the linguistic organisation of the narrative when narrating these actions and in the child's ability to designate the consequences (success or failure) for each of these actions (e.g., the boy looks for the frog inside the boot, but it was not there).

When narrating the actions that constitute the plot, the younger children used statements with a simpler structure and more restricted in the use of cohesive elements to demarcate temporality and causality between the narrated actions. The children in the 5-year-old group presented more descriptive narratives and the main temporal cohesive element used was “there” and “after” (e.g., the boy called the frog there he looked for it in a hole). Such elements have also been described as part of the cohesive resources used by children of narrative schema acquisition age^(22)^.

In turn, students aged 8 and over were able to use syntactically more complex statements with cohesive elements that demarcated the causality between the actions narrated, also exposing a consequence for each attempt to solve the problem (“the boy called the frog loudly, but he did not hear the frog. Then he saw a hole very close to there and decided to see if the frog was inside, but he didn't find the frog there either). Causal relations represent a central component in the representational model of narrative schemata, and mastery of this conceptualisation is an important marker of narrative development^(23)^.

A characteristic observed in the oral narrative of the group from the age of 10 was the ability of the students to linguistically and temporally organise a scene in actions experienced by more than one character (simultaneity of actions) and to retrieve information from previous scenes (previous sheet) to give meaning and continuity to the scene being narrated, without necessarily going back to the previous page (e.g., “the boy was looking for the frog in a hole inside the tree. Meanwhile, the dog was still standing in the tree that had that bees’ hive, and suddenly the hive fell to the ground and the bees started coming out in droves to sting the dog. The boy was so busy looking for the frog in the hole in the tree that he didn't even realise what was happening to the puppy). Another characteristic was the ability to express the simultaneity of events, which is indicative of the ability to use more complex mental schemas. The ability to represent temporal schemas is honed throughout the child's cognitive and language development and is what also allows the elaboration of more elaborate and complex narratives, consisting of multiple episodes^(24)^.

Still regarding the actions, it was also proposed to score the actions that represent the challenges of the story (“challenges” item). The challenges represent the obstacles faced by the main character in favour of solving the problem. These obstacles allow the narrator to create sub-episodes within the narrative, configuring a more complex narrative structure^(15)^, which justifies the fact that we found a higher score in this item for groups aged 10 and over.

Regarding the conventional linguistic elements for opening and closing the story, no statistically significant differences were found between the age groups investigated. It was possible to observe that older students, especially those aged 10 and over, used more efficient ways to linguistically organise the information in the scenario to introduce it without the need for a linguistic element to demarcate this opening (e.g., John and his dog were in the room watching the pet frog inside the glass jar”). On the other hand, the younger ones, mainly five-year-old preschoolers and 6-7-year-old schoolchildren, often introduced the story by identifying the characters and the objects that made up the scene (e.g., The boy was sitting with his dog. There was a frog in the jar”).

Another characteristic observed more frequently in the group from 8-9 years old was the beginning of the story by the initial event. In this regard, it should be noted that the narration was preceded by the visual reading of the book so that the children had the opportunity to mentally construct the story to be subsequently narrated orally. At the time of visual reading, linguistic markers are not necessary. The identification of the scene that informs the problem of the story and that brings the initial event (complication) is a fundamental element since it is the initial event that determines the understanding of a whole sequence of images a posteriori^(25)^. When the child was asked to narrate the story orally, even though the book was used as a support, this prior mental representation may have favoured the suppression of the typical linguistic markers of opening story narratives, causing the child to be directed to the starting point of the story.

An interesting finding was how preschoolers and schoolchildren used linguistic markers of closure, depending on the type of outcome presented (problem-solving). In narratives with an outcome closely related to the problem, observed from the age of 8-9, schoolchildren were able to use these markers during a more elaborate linguistic organisation without a temporal demarcation in the statement to signal the end of the story (“John took one of the frog's little children with him and everyone was happy”), while preschoolers used these linguistic markers separately from the outcome, as an explicit temporal marking of the end of the story (e.g. “The boy found the frog. And the end”), without jeopardising the coherent ending of the story.

Although unusual, the coherent opening or closing of a story can come without its conventional linguistic markers, without compromising comprehensibility by the listener, as in the aforementioned example, which may justify the fact that many participants did not use such linguistic elements in the narrative but achieved higher levels of overall coherence. It is worth mentioning that the classification system of the level of coherence proposed by Spinillo and Martins^(21)^, adopted in this study, considers as part of the analysis criteria the factor “comprehensibility”, *i.e*., whether the opening or closing is closely related to the narrated events. Although the scoring system mentions conventional linguistic markers as part of the characteristics to be observed in the narration, they do not influence the decision of the classification of the level of coherence.

In addition to investigating performance, the present study aimed to analyse the effect of age on the narrative score established through the ProNOH in macrostructure aspects. The results showed that the narrative score of the five-year-old group was lower and statistically different from the six-year-old and seven-year-old groups. The seven-year-old group had lower and statistically different means compared to the means of the eight- and nine-year-old groups and the nine-year-old group had lower and statistically different means from the 10- and 11-year-old groups, which, in turn, did not present a statistically significant difference between them. The difference found occurred in the comparison between the average of the 11-year-old groups (lower) concerning the 12-year-old group. Thus, it is noted that the narrative score was a more sensitive measure to differentiate narrative performance from five to six years old, from seven to eight years old, from nine to 10, and from 11 to 12 years old.

The most significant difference was found between the narrative scores of the borderline age groups from five (preschool) to six years old, which denotes important gains in the mastery and organisation of the structural elements typical of story grammar in this age group, also enabling more complex levels of narrative from the early school years onwards^(12,20,21)^. The findings in the literature show divergent data concerning the presence or absence of more marked differences in the mastery and use of the typical macrostructure components of story grammar among children aged five to 12 years, but there is a certain consensus that the preschool period, between four and five years old, is the one that presents the most significant changes^(26)^.

In the analyses carried out on the correlation between the narrative score, the level of coherence, and age, the narrative score tended to increase with increasing chronological age, especially from five to six years old, when level 2 of coherence became predominant. Successively, level 3 becomes predominant from the age group of seven until level 4, which is the most complex level of global coherence of the story, becoming predominant from 11 and 12 years old.

A similar finding was found for the level of global coherence of the story since older students, also with more education level, presented more complex levels of story organisation, corroborating previous studies that highlighted that age and education are important factors in learning and mastering the narrative story scheme^(20,21,27,28)^.

The correlation analysis conducted to investigate whether the narrative score would correlate with the level of overall story coherence showed a positive relationship between these two measures, indicating that the highest narrative scores were also those with the highest levels of coherence (Level 3 and Level 4). The system for analysing the level of overall story coherence was proposed in this study as a complementary research method to verify whether the presence of structural and linguistic story elements, represented by a narrative score, could also reflect the organisation of these elements in the narrative.

It is well established in the literature that the typical structural elements of the narrative story schema are relevant as part of the coherence factors of the narrative and that, when viewed together with the organisation of the narrated events, this set of factors plays an important role in establishing overall coherence^(21,26,27,29,30)^.

Evidently, the set of elements listed in the ProNOH does not exhaust all the aspects inherent to the story narrative scheme inscribed in the macrostructure dimension; therefore, other forms of analysis are necessary that allow the evaluator to access information of another magnitude about the story narrative.

This study has limitations that should be considered, including the number of subjects in the sample and the wide age range studied, which implies the need to expand the sample so that reference values can be provided for the macrostructure aspects of the ProNOH. Therefore, the scores presented here constitute an important parameter for the use of the ProNOH but still require further studies.

Despite the limitations pointed out, this study contributes to an area still lacking in Brazil, which is the availability of tools with collection criteria and a scoring system of oral storytelling for use in the context of narrative language research in the period of language acquisition and development.

## CONCLUSION

The proposed scoring system proved to be a useful tool to investigate the repertoire of typical story elements in oral storytelling of children aged between five and 12 years, since the overall narrative score measured and differentiated the performance of children belonging to different age groups (5 years<6-7 years<8-9 years<10-11 years<12 years).

Although the use of typical story elements in the narration does not ensure the establishment of a coherent story, it can be said that the analysis of the narrative score including the story elements used seems to give important directions to the evaluator about the coherence of the story, since the analyses of the data pointed to the positive relationship between the results coming from the two systems of analysis of the narration - global score and level of global coherence of the story.

Indeed, given the complexity of the task of writing a story and considering the number of variables that can influence performance in this task; social, educational, and neurobiological, it is known that a single instrument as a means of measurement does not exhaust all the aspects that subsidise this cognitive, linguistic and social activity in all its dimensions; and it does not exhaust all the possibilities of analysis that are possible through a sample of oral narrative.
