# Supplementary material for: Performance of preschool and schoolchildren on the ProNOH protocol: macrostructure aspects
Source: Codas. 2023 Nov 20;36(1):e20220245. doi: 10.1590/2317-1782/20232022245en (PMC10715574; doi:10.1590/2317-1782/20232022245en)
Supplement: Translation

# Translation: Desempenho de pré-escolares e escolares no protocolo ProNOH: aspectos macroestruturais

**DOI:** 10.1590/2317-1782/20232022245pt

**Authors:** Natalia Freitas Rossi, Ana Carolina Xavier, Kriscia Gobi Rosa, Célia Maria Giacheti

**Affiliations:** 1 Programa de Pós-graduação em Fonoaudiologia, Faculdade de Filosofia e Ciências de Marília, Universidade Estadual Paulista “Júlio de Mesquita Filho” – UNESP - Marília (SP), Brasil.; 2 Instituto Nacional de Ciência e Tecnologia sobre Comportamento, Cognição e Ensino – INCT-ECCE, Universidade Federal de São Carlos – UFSCar - São Carlos (SP), Brasil.; 3 Departamento de Fonoaudiologia, Faculdade de Filosofia e Ciências de Marília, Universidade Estadual Paulista “Júlio de Mesquita Filho” – UNESP - Marília (SP), Brasil.

**Keywords:** Narração, Linguagem, Desenvolvimento Infantil, Testes de Linguagem, Estudantes

## Abstract

**Objetivo:**

Investigar se o escore narrativo obtido por meio da aplicação do “Protocolo de Avaliação da Narrativa Oral de História (ProNOH)” permite discriminar os grupos etários estudados, bem como sua relação com o nível de coerência global da história.

**Método:**

Participaram 97 pré-escolares e escolares com desenvolvimento típico de linguagem, entre 5 e 12 anos, ambos os sexos que frequentavam escola pública. Foi aplicado o “Protocolo de Avaliação da Narrativa Oral de História (ProNOH)” e calculado o escore narrativo na dimensão macroestrutural a partir dos elementos estruturais previstos no modelo da gramática de história. Esses mesmos elementos foram usados para obter o nível de coerência global da história, segundo proposta de análise de Spinillo e Martins (1997).

**Resultados:**

Diferença estatisticamente significante foi encontrada entre os grupos etários, principalmente entre as idades fronteiriças de 5-6 anos, 7-8 anos, 9-10 anos e 11-12 anos. Correlação positiva e estatisticamente significante foi encontrada com a idade e o escore narrativo e a coerência global, bem como entre o escore narrativo e o nível de coerência global.

**Conclusão:**

O protocolo mostrou ser útil para identificar o repertório de elementos típicos da gramática de história na narrativa oral como uma medida objetiva que diferenciou a narrativa oral de grupos etários. Os resultados indicaram ainda que o escore narrativo pode ser uma medida norteadora do nível de coerência da história, apesar deste valor não substituir uma análise específica.

## INTRODUÇÃO

A narrativa oral é uma das habilidades discursivas e representa um dos níveis mais complexos de organização da linguagem com esquema cognitivo próprio de representação mental, com regras e elementos estruturais que são peculiares e conferem especificidades que permitem distinguir uma conversa de uma narrativa, bem como distinguir os diferentes tipos de narrativa; autobiográfica ou história^(1)^.

A narrativa do tipo história é constituída por elementos estruturais típicos da gramática de história, os quais são considerados parte dos aspectos macroestruturais da narrativa. A dimensão da macroestrutura pode ser considerada o componente cognitivo, de ordem executiva e que é responsável pela organização geral do enredo, pela sustentação do tema e pela manutenção de informações ao longo da narrativa, a fim de constituir um enredo lógico e coerente Por sua vez, a dimensão microestrutural pode ser considerada a dimensão de natureza linguística, que compreende a fonologia das palavras, a organização e complexidade sintática dos enunciados, o vocabulário, bem como o uso de recursos coesivos. Ambos os aspectos, macro e microestruturais, têm sido adotados em estudos como indicadores do desenvolvimento da habilidade para narrar histórias^(2-4)^.

Crianças que apresentam desvio no desenvolvimento da linguagem podem apresentar tanto dificuldades para compreender quanto para narrar histórias^(5,6)^. Os efeitos dos prejuízos cognitivo-linguísticos sobre as habilidades narrativas nessas crianças são descritos como heterogêneos e variáveis a depender da etiologia e de outros fatores relacionados^(7)^

No Transtorno do Desenvolvimento da Linguagem de origem primária as alterações parecem estar mais centradas nos aspectos microestruturais da narrativa^(5)^. Por outro lado, os prejuízos na macroestrutura da narrativa têm sido bastante explícitos nos casos em que as alterações de linguagem estão associadas à síndromes genéticas que cursam com Transtorno do Desenvolvimento Intelectual, bem como no Transtorno do Espectro Autista, conforme apontado em estudo de revisão^(7)^ e no Transtorno do Déficit de Atenção e Hiperatividade^(8,9).^

A narrativa oral tem sido considerada um elo importante entre as habilidades da linguagem oral e da escrita devido ao compartilhamento de processos cognitivos que subsidiam as modalidades de linguagem, a exemplo das funções executivas^(10)^.

Para além das funções executivas, a tarefa de narrativa oral de história reflete ainda a integração dos componentes estruturais da linguagem (e.g., morfossintaxe, fonologia) e de conteúdo (semântica), os quais são aplicados na comunicação funcional (pragmática), gerenciados pelos elementos estruturais típicos da gramática de história^(10)^.

A respeito dos métodos de investigação do desempenho na narrativa oral é possível encontrar na literatura diferentes propostas de coleta e de análise. O uso de uma sequência de imagens, para eliciar histórias, principalmente por meio de livros é um recurso considerado favorável, uma vez que auxilia na elaboração do esquema narrativo de história e permite ao avaliador; em alguma medida, certo controle do conteúdo que está sendo narrado, o que não é possível nas narrativas pessoais^(11,12)^.

Dentre os livros-imagem mais citados na literatura como recurso para eliciar narrativa o livro “*Frog, where are you*?”^(13)^ é amplamente referenciado. Sua estrutura é vantajosa para a realização de estudos transculturais, uma vez que dispõe apenas de ilustrações, sem escrita, com nível de complexidade suficiente para uma análise de aspectos importantes nos estudos sobre narrativa, como as relações temporais, causais e espaciais entre eventos^(14)^. O livro “*Frog, where are you*?”^(13)^ possui organização interna que favorece a organização de níveis complexos do esquema narrativo de história e permite analisar diferentes faixas etárias, incluindo adultos^(14,15)^.

Este livro^(13)^ tem sido utilizado tanto em estudos conduzidos com indivíduos com desenvolvimento típico quanto atípico de linguagem^(4,16)^ e também utilizado como parte de protocolos para propor um sistema de análise do desempenho narrativo, a partir dos elementos macro e microestruturais^(17-20)^.

A escassez de imagens sequenciais específicas e padronizadas para investigar a narrativa oral de história é uma realidade no cenário clínico e de pesquisa brasileiro. Neste contexto, o “Protocolo de Avaliação da Narrativa Oral de História (ProNOH)”^(19)^ foi desenvolvido como uma proposta sistemática para a análise dos aspectos macro e microestruturais da narrativa eliciada a partir do livro “*Frog, where are you*?”^(13)^. O protocolo apresenta instruções para análise do desempenho narrativo por meio de pontuação atribuída pelo avaliador a partir dos elementos típicos de história, que permite determinar o escore narrativo, que é gerado a partir da dimensão macroestrutural e critérios relativos aos aspectos microestruturais, constituindo assim uma ferramenta que pode ser utilizada tanto para fins de avaliação, quanto para monitoramento terapêutico (evolução) das habilidades narrativas^(19)^.

Sendo assim, o presente estudo propôs investigar se o escore narrativo obtido por meio da aplicação do “Protocolo de Avaliação da Narrativa Oral de História (ProNOH)”^(19)^ permite discriminar os grupos etários estudados, bem como sua relação com o nível de coerência global da história.

## MÉTODO

Este estudo faz parte de um projeto mais amplo aprovado pelo Comitê de Ética em Pesquisa da Universidade Estadual Paulista “Júlio de Mesquita Filho” (UNESP) – Câmpus de Marília (processo nº 1105/2014; 5.391.347). Os pais e/ou responsáveis autorizaram a participação das crianças por meio da assinatura do Termo de Consentimento Livre e Esclarecido,

A amostra foi constituída por 97 participantes (47 do sexo masculino e 50 do sexo feminino) com idade entre 5 e 12 anos (M=8,46, DP=2,34), frequentando a Educação Infantil até o Ensino Fundamental I (1^a^ a 6^a^ série) de três escolas públicas de um município do interior do Estado de São Paulo.

Os critérios de inclusão adotados foram: (a) não apresentar história prévia e/ou alteração persistente de linguagem/aprendizagem, bem como condição médica sindrômica, transtornos psiquiátricos e/ou neurológicos; (b) não apresentar alterações sensoriais (auditiva e/ou visual), (c) estar frequentando o ensino público e regular (d) ter sido indicado pelo professor como aluno com desempenho adequado para a escolaridade. Na Tabela 1 estão sumarizados a distribuição quanto à frequência por grupo etário e sexo dos participantes.

**Tabela 1:** Distribuição da amostra nos grupos etários

Grupo Etário	Frequência	Sexo (%)	
		Masculino	Feminino
5	13	4 (30,7)	9 (69,2)
6	12	9 (75,0)	3 (25,0)
7	13	8 (61,5)	5 (38,5)
8	12	7 (58,3)	5 (41,7)
9	11	3 (27,3)	8 (72,7)
10	11	3 (27,3)	8 (72,7)
11	12	5 (41,7)	7 (58,3)
12	13	8 (61,5)	5 (38,5)

A narrativa oral foi coletada e analisada quanto aos aspectos macroestruturais utilizando o livro *“Frog, where are you?*”^(13)^ e segundo instruções e critérios de análise e pontuação propostos no “Protocolo de Avaliação da Narrativa Oral de História - ProNOH”^(19)^.

O tempo médio despendido para utilização do protocolo foi de: (a) 3,5 a 5,0 minutos para a narração, (b) 50 minutos para transcrição da narrativa e (c) 30 minutos para atribuição dos pontuação e soma dos escores parciais e globais da narrativa.

Para obter o escore narrativo atribuiu-se pontuação segundo a presença de informações distribuídas em 5 categorias estruturais de história: cenário, tema, enredo, desafios e resolução, conforme proposto por Rossi, Rosa e Giacheti^(19)^ e o escore parcial e global foi determinado.

Para classificação do nível de coerência global da história, adotou-se a proposta de Spinillo e Martins (1997)^(21)^ para análise da coerência global da história, e considera como indicadores de coerência os elementos estruturais, os mesmos anteriormente identificados para estabelecer o escore narrativo pelo protocolo ProNOH. Os itens analisados para estabelecimento do nível de coerência foram: a manutenção dos personagens ao longo da narrativa; a manutenção do tema ao redor do evento principal e eventos secundários que se articulam ao evento principal e a relação entre os eventos narrados conectados entre si e aos personagens, com a presença de um desfecho que finaliza e conclui a história com relação ao evento principal. A partir desses fatores, as histórias foram classificadas em quatro níveis crescentes de complexidade (Nível 1, Nível 2, Nível 3 e Nível 4), que informam o grau de coerência global da história.

### Análise estatística

Foi realizada análise estatística descritiva para obter os valores da média, desvio padrão e percentis 25, 50 e 75 do escore narrativo parcial e global. Para o caso em que o teste de normalidade *Shapiro Wilk* indicou que os dados apresentavam aderência à normalidade prosseguiu-se com o teste de igualdade das médias para verificar possíveis diferenças entre as médias por meio da análise de variância *ANOVA* com um fator com várias comparações. A análise post-hoc de comparações múltiplas foi conduzida com o teste de *Tukey* quando o resultado da Anova indicou significância estatística (p<0,05).

O teste estatístico de correlação de Pearson foi usado para investigar possível correlação do escore narrativo (escore total) com a idade e o nível de coerência global da história.

O nível de significância adotado no estudo foi de 0,05%. A análise dos dados foi realizada utilizando o programa Minitab 1.6.

## RESULTADOS

Na Tabela 2 estão as médias, desvios-padrão e percentis do escore narrativo dos grupos etários de 5 a 12 anos estabelecidos a partir do ProNOH nos aspectos macroestruturais.

**Tabela 2:** Médias, desvios-padrão e percentis dos grupos etários no ProNOH-aspectos macroestruturais

		Idade							
		5 anos	6 anos	7 anos	8 anos	9 anos	10 anos	11 anos	12 anos
Cenário	Média	2,5	3,4	4,0	4,6	4,6	5,2	5,7	6,0
	DP	0,5	0,5	0,7	0,9	1,0	0,7	0,9	1,0
	Percentil 25	2,0	3,0	3,5	4,0	4,0	5,0	5,0	5,0
	Percentil 50	2,0	3,0	4,0	4,5	4,0	5,0	5,0	6,0
	Percentil 75	3,0	4,0	4,5	5,0	5,0	6,0	6,7	7,0
Tema	Média	0,9	2,0	2,0	2,0	2,0	2,0	2,0	2,0
	DP	0,6	0,0	0,0	0,0	0,0	0,0	0,0	0,0
	Percentil 25	0,5	2,0	2,0	2,0	2,0	2,0	2,0	2,0
	Percentil 50	1,0	2,0	2,0	2,0	2,0	2,0	2,0	2,0
	Percentil 75	1,0	2,0	2,0	2,0	2,0	2,0	2,0	2,0
Enredo	Média	1,6	3,7	4,7	4,7	5,2	5,7	5,5	6,3
	DP	0,6	0,9	0,7	0,8	1,2	0,6	0,5	1,1
	Percentil 25	1,0	3,0	4,0	4,0	4,0	5,0	5,0	5,5
	Percentil 50	2,0	4,0	5,0	4,5	6,0	6,0	5,5	6,0
	Percentil 75	2,0	4,0	5,0	5,0	6,0	6,0	6,0	7,0
Resolução	Média	0,8	1,1	1,1	1,3	1,6	2,0	2,0	2,0
	DP	0,4	0,3	0,4	0,5	0,5	0,0	0,0	0,0
	Percentil 25	1,0	1,0	1,0	1,0	1,0	2,0	2,0	2,0
	Percentil 50	1,0	1,0	1,0	1,0	2,0	2,0	2,0	2,0
	Percentil 75	1,0	1,0	1,0	2,0	2,0	2,0	2,0	2,0
Desafios	Média	2,2	4,8	4,2	5,4	5,4	6,0	6,0	7,5
	DP	0,4	1,0	0,7	0,9	1,1	0,8	0,9	0,9
	Percentil 25	2,0	4,0	4,0	5,0	4,0	5,0	5,0	6,0
	Percentil 50	2,0	4,5	4,0	5,5	6,0	6,0	6,0	8,0
	Percentil 75	2,5	5,7	5,0	6,0	6,0	7,0	7,0	8,0
Marcadores Linguísticos	Média	0,38	0,41	0,69	1,16	0,45	0,81	0,75	0,4
	DP	0,50	0,51	0,63	0,83	0,68	0,60	0,45	0,6
	Percentil 25	0,0	0,0	0,0	0,25	0,0	0,0	0,25	0,0
	Percentil 50	0,0	0,0	1,0	1,0	0,0	1,0	1,0	0,0
	Percentil 75	1,0	1,0	1,0	2,0	1,0	1,0	1,0	1,0
Total (Escore Narrativo)	Média	8,5	15,4	16,8	19,2	19,4	21,8	21,9	24,1
	DP	1,8	1,8	1,3	1,4	2,4	1,6	1,5	2,2
	Percentil 25	7,0	14,0	16,0	18,0	18,0	21,0	21,0	22,0
	Percentil 50	8,0	15,0	16,0	19,0	18,0	22,0	21,5	23,0
	Percentil 75	10,0	16,7	18,0	20,0	21,0	23,0	23,7	26,0

Legenda: DP = Desvio padrão

Na comparação das médias do escore narrativo nos grupos etários por meio da Anova foi encontrada diferença estatisticamente significante (F=95,21; p<0,000). Na análise post-hoc com o teste de Tukey verificou-se diferença estatisticamente significante entre as idades fronteiriças de 5-6 anos, 7-8 anos, 9-10 anos e 11-12 anos, com as seguintes representações de médias: 5 anos < 6 anos = 7 anos < 8 anos = 9 anos < 10 anos = 11 anos > 12 anos (Figura 1).

**Figura 1:** Escore narrativo (escore total) no ProNOH-aspectos macroestruturais nos grupos etários.

Legenda: (*) p<0,05. Análise post-hoc pelo teste de Tukey

A distribuição da frequência da classificação do nível de coerência global da história (Tabela 3) indicou que o nível de coerência mais baixo (Nível 1) somente foi encontrado no grupo etário de 5 anos. A maioria dos participantes entre 6 e 7 anos apresentou nível 3 de coerência. O grupo de 8 anos ficou igualmente dividido entre o nível 3 e 4 e acima dos 9 anos a maioria apresentou nível 4 de coerência global.

**Tabela 3:** Frequência dos níveis de coerência encontrados nos grupos etários

**Grupo Etário**	**Nível de Coerência**			
	**Nível 1**	**Nível 2**	**Nível 3**	**Nível 4**
5 anos (N=14)	11	3	0	0
6 anos (N=12)	0	2	10	0
7 anos (N=13)	0	0	13	0
8 anos (N=12)	0	0	8	4
9 anos (N=11)	0	0	4	7
10 anos (N=11)	0	0	2	9
11 anos (N=12)	0	0	1	11
12 anos (N=13)	0	0	0	13

Por meio do teste de correlação de Pearson foi encontrada correlação estatisticamente significante e positiva entre idade e o escore narrativo, (r=0,880 e p=0,000) e entre a idade e o nível de coerência global da narrativa (r=0,786, p=<0,001). A Figura 2 mostra a distribuição do escore narrativo e nível de coerência em função da idade.

**Figura 2:** Distribuição do escore narrativo e nível de coerência da história em função da idade dos participantes

Legenda: (*) p<0,05. Teste de correlação de Pearson

Também por meio do teste de correlação de Pearson foi encontrada correlação estatisticamente significante e positiva entre o escore narrativo e o nível de coerência global da história (r=0,888; p=0,000). A Figura 3 mostra a distribuição do escore narrativo em função do nível de coerência global da história dos participantes.

**Figura 3:** Distribuição do escore narrativo em função do nível de coerência global da história dos participantes

## DISCUSSÃO

Neste estudo foi proposto investigar se o escore narrativo obtido por meio da aplicação do “Protocolo de Avaliação da Narrativa Oral de História (ProNOH)”^(19)^ permitiria discriminar os grupos etários estudados, bem como sua relação com o nível de coerência global da história. O referido protocolo foi proposto para sistematização do recurso de elicitação da narrativa oral de história e análise dos elementos de história, propondo assim um sistema de pontuação que permitisse medir de forma objetiva o desempenho na narrativa oral de história a partir dos aspectos macro e microestruturais. Neste estudo os dados obtidos recaem especificamente sobre os aspectos macroestruturais.

A respeito do desempenho dos pré-escolares e escolares distribuídos nas faixas etárias de 5 a 12 anos, verificou-se que no elemento “cenário” a atribuição de nome próprio aos personagens (menino, cachorro e sapo) foi mais comum nas histórias narradas pelos escolares a partir dos 8-9 anos de idade. Também as crianças mais novas abaixo de 8 anos, principalmente os pré-escolares apresentaram mais dificuldade para perceber a existência de uma passagem temporal na história que é representada pelos por vários elementos gráficos como; a “lua”, que pode ser vista pela janela do quarto (cenas 1 e 2), o menino e o cachorro dormindo na cama (cena 2) e a ausência do elemento “lua” com a claridade entrando no quarto (cena 3). Nota-se na ilustração da cena 3 que a claridade é representada com a diminuição do sombreado na parede em que está disposta a janela.

Em relação ao item “tema”, verificou-se que apesar das crianças do grupo 6-7 anos serem capazes de identificar a existência de um problema ou complicação na história, explicitando o evento inicial, elas tiveram dificuldades para mencionar a existência de um plano interno do personagem principal (decisão do menino em procurar o sapo) para resolver o problema. A proposição de um plano de ação, bem como o uso de elementos linguísticos que deixam explícito esse plano (“o menino *decidiu* procurar o sapo”) esteve mais presente nas narrativas dos grupos 8-9 anos e, principalmente, a partir dos 10 anos.

A sucessão de ações temporalmente organizadas (representadas nas cenas 4 a 18 do livro) que mostram as várias tentativas do personagem resolver o problema constituem o enredo da narrativa. Na análise desse item, foi possível observar que o número de ações narradas pelas crianças foi diferente nos grupos etários. Qualitativamente também foi possível identificar diferenças entre os grupos. As principais diferenças foram vistas na organização linguística da narrativa, ao narrar essas ações e também na capacidade da criança designar as consequências (sucesso ou insucesso) para cada uma dessas ações (e.g., o menino procuro pelo sapo dentro da bota, mas ele não estava lá).

Ao narrar as ações que constituem o enredo, as crianças mais novas utilizaram enunciados com estrutura mais simples e também mais restritos quanto ao uso de elementos coesivos para demarcar temporalidade e causalidade entre as ações narradas. As crianças do grupo de 5 anos apresentaram narrativas mais descritivas e o principal elemento de coesão temporal utilizado foi “ai” e “depois” (e.g., o menino chamou o sapo ai ele procurou ele num buraco). Tais elementos também foram descritos como parte dos recursos coesivos utilizados por crianças em idade de aquisição de esquemas narrativos^(22)^.

Por sua vez, os escolares a partir dos 8 anos foram capazes de utilizar enunciados sintaticamente mais complexos com elementos coesivos que demarcavam a causalidade entre as ações narradas, expondo também uma consequência para cada tentativa de resolver o problema (“o menino chamou o sapo bem alto, mas ele não escutou o sapo. Então ele viu um buraco bem perto dali e resolveu ver se o sapo estava lá dentro, mas ele também não achou o sapo ali). As relações causais representam um componente central no modelo de representação de esquemas narrativos, sendo o domínio dessa concepção um importante marcador do desenvolvimental da narrativa^(23)^.

Uma característica observada na narrativa oral do grupo a partir dos 10 anos foi a capacidade que os escolares apresentaram para organizar linguisticamente e temporalmente uma cena em ações vividas por mais de um personagem (simultaneidade de ações) e de resgatar informações das cenas anteriores (folha anterior) para dar sentido e continuidade a cena que estava sendo narrada, sem necessariamente voltar à página anterior (e.g., “o menino estava procurando o sapo num buraco dentro da árvore. Enquanto isso, o cachorro continuava em pé na árvore que tinha aquela colmeia de abelhas e de repente a colmeia caiu no chão e as abelhas começaram a sair aos montes para picar o cachorro. O menino estava tão ocupado procurando o sapo no buraco da árvore que ele nem se tocou o que estava acontecendo com o cachorrinho). Outra característica foi a capacidade de expressar a simultaneidade de eventos, o que é indicativo da capacidade para uso de esquemas mentais mais complexos A habilidade para representar esquemas temporais é lapidado ao longo do desenvolvimento cognitivo e de linguagem da criança e é o que permite também a elaboração de narrativas mais elaboradas e complexas, constituídas por múltiplos episódios^(24)^.

Ainda no que diz respeito às ações, também foi proposto pontuar as ações que representam os desafios da história (item “desafios”). Os desafios representam os obstáculos enfrentados pelo personagem principal em prol da resolução do problema. Esses obstáculos permitem ao narrador criar sub-episódios dentro da narrativa, configurando uma estrutura narrativa mais complexa^(15)^, o que justifica o fato de termos encontrado maior pontuação neste item para os grupos a partir dos 10 anos.

A respeito dos elementos linguísticos convencionais de abertura e fechamento de história, conforme apresentado não foram encontradas diferenças estatisticamente significantes entre os grupos etários investigados. Foi possível observar que os escolares mais velhos, principalmente a partir dos 10 anos, utilizaram formas mais eficientes para organizar linguisticamente as informações do cenário de modo a introduzi-las sem que fosse necessário um elemento linguístico para demarcar essa abertura (e.g., João e o seu cachorro estavam no quarto observando o sapinho de estimação dentro do pote de vidro”). Por outro lado, os mais jovens, principalmente os pré-escolares de cinco anos e escolares de 6-7 anos, frequentemente introduziram a história com a identificação dos personagens e dos objetos que compunham a cena (e.g., o menino estava sentado com o seu cão. Tinha um sapo no pote”).

Outra característica observada mais frequentemente no grupo a partir dos 8-9 anos foi o início da história pelo evento inicial. A esse respeito é preciso ponderar que a narração era precedida pela leitura visual do livro, de modo que as crianças tinham a oportunidade de construir mentalmente a história a ser posteriormente narrada oralmente. No momento da leitura visual, os marcadores linguísticos não são necessários. Já a identificação da cena que informa o problema da história e que traz o evento inicial (complicação) é um elemento fundamental, uma vez que é o evento inicial que determina a compreensão de toda uma sequência de imagens a posteriori^(25)^. Na medida em que a criança foi solicitada a narrar oralmente a história, ainda que o livro tenha sido utilizado como apoio, essa representação mental prévia pode ter favorecido a supressão dos marcadores linguísticos típicos de abertura de narrativas de história, fazendo com que a criança fosse direcionada para o ponto de partida da história.

Um dado interessante foi a forma como os pré-escolares e escolares utilizaram os marcadores linguísticos de fechamento, mediante o tipo de desfecho apresentado (resolução do problema). Nas narrativas com desfecho estreitamente relacionado com o problema, observado a partir dos 8-9 anos, os escolares eram capazes de utilizar esses marcadores em meio a uma organização linguística mais elaborada sem que houvesse uma demarcação temporal no enunciado para sinalizar o fim da história (“João levou um dos filhinhos do sapo com ele e todos ficaram felizes”) ao passo que os pré-escolares utilizavam esses marcadores linguísticos separadamente do desfecho, como uma marcação temporal explícita do encerramento da história (e.g., “O menino achou o sapo. E fim), sem que isso comprometesse o final coerente da história.

Apesar de pouco usual, a abertura ou encerramento coerente de uma história, ao que parece, pode vir desprovido dos seus marcadores linguísticos convencionais, sem que isso comprometa a compreensibilidade por parte do ouvinte, como o exemplo supracitado, o que pode justificar o fato de muitos participantes não terem utilizado tais elementos linguísticos na narrativa, mas terem alcançado níveis mais altos de coerência global. É válido mencionar que o sistema de classificação do nível de coerência proposto por Spinillo e Martins^(21)^, adotado neste estudo, considera como parte dos critérios de análise o fator “compreensibilidade”, ou seja, se a abertura ou o fechamento estão em estreita relação com os eventos narrados. Embora o sistema de pontuação mencione os marcadores linguísticos convencionais como parte das características a serem observadas na narração, os mesmos não influenciam na decisão da classificação do nível de coerência.

Para além da investigação do desempenho, o presente estudo propôs analisar o efeito da idade sobre o escore narrativo estabelecido por meio do ProNOH nos aspectos macroestruturais. Os resultados permitiram verificar que o escore narrativo do grupo de 5 anos foi inferior e estatisticamente diferente do grupo de 6 anos e 7 anos. O grupo de 7 anos teve média inferior e estatisticamente diferente comparada às médias dos grupos 8 e 9 anos, e o grupo de 9 anos teve média inferiores e estatisticamente diferentes do grupo 10 e 11 anos, que por sua vez não apresentaram diferença estatisticamente significante entre si. A diferença encontrada ocorreu na comparação entre a média dos grupos de 11 anos (inferior) em relação ao grupo de 12 anos. Assim, nota-se que o escore narrativo foi uma medida mais sensível para diferenciar o desempenho narrativo de cinco para seis anos, de sete para oito anos, de nove para 10 e de 11 para 12 anos.

A diferença mais expressiva foi encontrada entre os escores narrativos dos grupos etários fronteiriços de 5 anos (pré-escolar) para 6 anos, o que denota ganhos importantes no domínio e organização dos elementos estruturais típicos da gramática de história nesta faixa etária, possibilitando também níveis mais complexos de narrativa a partir dos primeiros anos escolares^(12,20,21)^.^.^ Os achados da literatura mostram dados divergentes quanto à presença ou não de diferenças mais marcantes no domínio e uso dos componentes macroestruturais típicos da gramática de história entre crianças de 5 a 12 anos de idade, mas há certo consenso que o período pré-escolar, entre 4 e 5 anos de idade, é o que apresenta mudanças mais significativas^(26)^

Fato este que, nas análises realizadas quanto à correlação entre o escore narrativo, o nível de coerência e a idade verificou-se que o escore narrativo tende a aumentar com o aumento da idade cronológica, principalmente dos 5 para os 6 anos, quando nível 2 de coerência passou a ser predominante utilizado. E sucessivamente, o nível 3 passa a ser predominante a partir do grupo de 7 anos até que o nível 4, que é o nível mais complexo de coerência global da história, passa a ser predominante a partir de 11 e 12 anos.

Achado semelhante foi encontrado para o nível de coerência global da história, uma vez que os escolares mais velhos, também com maior escolaridade, apresentaram níveis mais complexos de organização da história, corroborando estudos anteriores que ressaltaram que a idade e a escolaridade são fatores importantes no aprendizado e domínio do esquema narrativo de história^(20,21,27,28)^.

A análise de correlação realizada a fim de investigar se o escore narrativo apresentaria correlação com o nível de coerência global da história mostrou relação positiva entre essas duas medidas, indicando que os escores narrativos mais altos também foram as que apresentaram níveis mais altos de coerência (Nível 3 e Nível 4). O sistema de análise do nível de coerência global da história foi proposto neste estudo como um método de investigação complementar para verificar se a presença dos elementos estruturais e linguísticos de história, representados por um escore narrativo, poderia refletir também a organização desses elementos na narrativa.

É bem estabelecido na literatura que os elementos estruturais típicos do esquema narrativo de história são relevantes como parte dos fatores de coerência da narrativa e que, quando vistos conjuntamente com a organização dos eventos narrados, esse conjunto de fatores assumem importante papel no estabelecimento da coerência global^(21,26,27,29,30)^.

Evidentemente, o conjunto de elementos elencados no ProNOH não esgotam todos os aspectos inerentes ao esquema narrativo de história inscritos na dimensão macroestrutural sendo; portanto, necessárias outras formas de análise que permitam ao avaliador o acesso à informações de outra magnitude sobre a narrativa de história.

Este estudo apresenta limitações as quais devem ser consideradas, dentre elas o número de sujeitos que compôs a casuística e a faixa etária ampla estudada, o que implica na necessidade de ampliar a amostra para que possam ser fornecidos valores de referência para os aspectos macroestruturais do ProNOH. Portanto os escores aqui apresentados constituem um importante parâmetro para uso do ProNOH, mas que ainda requer estudos complementares.

Apesar das limitações apontadas, este estudo contribui para uma área ainda carente no Brasil que é a disponibilidade de ferramentas com critérios de coleta e sistema de pontuação da narrativa oral de história para fins de uso no contexto de investigação da linguagem narrativa no período de aquisição e desenvolvimento da linguagem.

## CONCLUSÃO

O sistema de pontuação proposto mostrou ser uma ferramenta útil para investigar o repertório de elementos típicos de história na narrativa oral de crianças com idade entre cinco e 12 anos, uma vez que o escore global da narrativa mediu e diferenciou o desempenho de crianças pertencentes a diferentes grupos etários (5 anos<6-7 anos<8-9 anos<10-11 anos<12 anos).

Embora, o uso dos elementos típicos de história na narração não assegure o estabelecimento de uma história coerente, pode-se dizer que a análise do escore narrativo incluindo os elementos de história utilizados parecem dar direcionamentos importantes para o avaliador sobre a coerência da história, uma vez que as análises dos dados apontaram para a relação positiva entre os resultados oriundos dos dois sistemas de análise da narração – escore global e nível de coerência global da história.

Evidentemente, frente a complexidade que representa a tarefa de elaborar uma história e considerando o número de variáveis que podem influenciar o desempenho nessa tarefa; de ordem social, educacional e neurobiológica, sabe-se que um único instrumento como meio de medida não esgota todos os aspectos que subsidiam esta atividade cognitiva, linguística e social em todas as suas dimensões; bem como, não esgota todas as possibilidades de análise que se é possível mediante uma amostra de narrativa oral.
